# Epilepsy

**Published:** 1890-04-19

**Authors:** 


### EPILEPSY.

In the *Boston Medical and Surgical Journal* last received, Dr. Pomroy gives his experience of the treatment of epilepsy by borax and bromide of potassium at the State Asylum for the Insane at Rhode Island. Twenty epileptics were placed on ten grains of borax three times daily. For a month previously these patients took no medicine, and a careful account of the epileptic seizures was kept. While the patients were taking the borax there was no diminution in the number of attacks. After an interval of a month the same epileptics were given twenty grains of bromide three times a-day. The number of attacks were diminished in nearly all cases, but the treatment had to be discontinued either on account of acne or the feeble condition of the patient. After the treatment was stopped the epileptic attacks became as frequent as before.

